# Methanol Dehydration to Dimethyl Ether on Zr-Loaded P-Containing Mesoporous Activated Carbon Catalysts

**DOI:** 10.3390/ma12132204

**Published:** 2019-07-09

**Authors:** José Palomo, José Rodríguez-Mirasol, Tomás Cordero

**Affiliations:** Chemical Engineering Department, Andalucía Tech, Universidad de Málaga, 29010 Málaga, Spain

**Keywords:** dimethyl ether, methanol dehydration, activated carbons, zirconium phosphates, phosphorus

## Abstract

Activated carbons have been prepared by the chemical activation of olive stones with phosphoric acid and loaded with Zr. The addition of Zr to the phosphorus-containing activated carbons resulted in the formation of zirconium phosphate surface groups. Gas phase methanol dehydration has been studied while using the prepared Zr-loaded P-containing activated carbons as catalysts. Carbon catalysts showed high steady-state methanol conversion values, which increased with Zr loading up to a limit that was related to P content. The selectivity towards dimethyl ether was higher than 95% for all Zr loadings. Zirconium phosphate species that were present on catalysts surface were responsible for the catalytic activity.

## 1. Introduction

Dimethyl ether (DME) is playing an important role due to its potential use as an alternative fuel in diesel engines. The use of this fuel produces lower NOx emissions and less engine noise when compared to traditional diesel fuels [1,2]. Moreover, this compound is used as a building block for many value-added chemicals, such as lower olefins, methyl acetate, and dimethyl sulphate [3,4]. Two alternatives can be used for DME production: (i) a two-step process (the so-called indirect process), which consists of one stage of methanol production from syngas while using a Cu/ZnO based catalyst [5,6,7], followed by a second stage of methanol dehydration over a solid acid catalyst [2,8,9,10]; (ii) a direct process, in which syngas is fed to a reactor that contains a bifunctional catalyst where the two aforementioned reactions take place [11,12,13,14]. The synthesis gas can be produced by partial oxidation or reforming of natural gas or by gasification of coal or biomass. In this sense, biomass represents a renewable carbon source, which would lead to a considerable reduction of greenhouse gas emissions in this process, promoting the transition to an energy system that reduces the dependence on fossil fuels in a scenario that simultaneously contemplates the scarcity of them, the growth of global demand and the impact of emissions on the environment.

The catalytic dehydration of methanol is currently the main source of DME production. In this sense, many studies have been carried out in order to obtain catalysts with a high activity and selectivity towards DME [15,16,17,18,19,20,21]. Among the studied catalysts, γ-Al_2_O_3_ and zeolites have been widely investigated. On one hand, γ-Al_2_O_3_ is considered to be the most efficient one, owing to its high mechanical resistance, excellent lifetime, and high selectivity towards DME [22]. However, there are some unsolved drawbacks, such as the preferential adsorption of water produced during the methanol dehydration reaction on the Lewis acid sites, reducing its catalytic activity that way. On the other hand, Zeolites (most often H-ZSM-5) are also commonly employed as catalysts for methanol dehydration [23]. These materials show high hydrothermal stability and high catalytic activity. Yet, the presence of strong acid sites yields the production of undesirable by-products and coke deposition, which lowers the productivity of DME. The acid strength of these materials must be reduced to avoid coke deposition and increase the selectivity towards DME [24].

Activated carbons that are used as catalysts and catalyst supports have received significant attention in the recent decades, owing to some great advantages, such as its high specific surface area, high thermal and chemical stability, and the possibility to modify its surface chemistry, tuning its acidity that way. These materials can be obtained from several kinds of lignocellulosic waste [25,26,27], producing economic as well as environmental advantages. However, the use of activated carbons as catalysts for methanol dehydration has scarcely been studied so far, despite its potential. Regarding this issue, Zawadzki et al. [28] pointed out that non-oxidized carbons are unreactive in the methanol decomposition reaction, observing certain activity only after oxidizing its surface by using gas oxygen. In this sense, some attempts have been made in order to modify the carbon surface by oxidation with chemical reagents, such as (NH_4_)_2_S_2_O_8_, H_2_SO_4_, and HNO_3_ [29,30]. However, the acid groups that were created by these means have a low thermal stability, which results in a fast deactivation when the reaction temperature is increased.

Our research group has reported several works on the preparation and characterization of biomass waste derived activated carbons by chemical activation with phosphoric acid [31,32,33]. The activation with phosphoric acid leads to the preparation of carbon materials, presenting oxygen-phosphorus surface groups that show a high thermal and chemical stability, remaining over the carbon surface after the washing step [34]. These phosphorus surface groups provide the activated carbons a high oxidation resistance and surface acidity, which makes them suitable for catalysis applications. According to this fact, these materials have been successfully used as catalyst in the decomposition of alcohols, mainly yielding dehydration products [35,36]. In this line, methanol dehydration has been achieved on these materials, obtaining a selectivity towards DME that is higher than 95% when air is used as a reaction atmosphere [37]. However, when they are applied as catalysts in a non-oxidizing atmosphere, they suffer from fast deactivation due to coke deposition, only exhibiting a residual methanol conversion that is lower than 10%. Therefore, these materials cannot be applied to industrial DME production routes, that is, indirect as well as direct DME synthesis processes.

In the light of the aforementioned statements, the use of activated carbons for feasible methanol dehydration applications is still a pending issue. In this work, the modification of phosphorus-containing olive stone-derived activated carbon with zirconium has been analyzed, with the aim of preparing a carbon-based catalyst with a high resistance to coke deactivation and a tailor-made acidity for the selective methanol dehydration to DME, which would bring high added-value to this agri-food industry waste.

## 2. Materials and Methods 

### 2.1. Preparation of Activated Carbon

Activated carbons were prepared by the chemical activation of olive stone, an agri-food industry waste product, with phosphoric acid. In this process, 10 g of biomass precursor, which Sociedad Cooperativa Andaluza Olivarera y Frutera San Isidro, Periana (Malaga, Spain) supplied, were impregnated with H_3_PO_4_ 85% (*w/w*) aqueous solution, at room temperature and then dried for 24 h at 60 °C, in a vacuum dryer. The impregnation ratio value (H_3_PO_4_/olive stone mass ratio) used in this case was 2. Once impregnated and dried, the substrate was activated in a conventional tubular furnace (Carbolite CTF 12/100/900, Thermo Fisher Scientific, Waltham, MA, USA) under continuous N_2_ flow (150 cm^3^/min. STP) at 800 °C for 2 h. The activated sample was cooled inside the furnace under the same N_2_ flow and then washed with distilled water at 60 °C until displaying a neutral pH and a negative phosphate analysis in the eluate [38]. The final activated carbon that was prepared following this procedure presented a yield of 39% based on the mass of dried olive stone and was denoted as ACP2800. 

An activated carbon was prepared by physical activation for comparative purposes. This process consisted of the carbonization of 15 g of the same carbon precursor, followed by a partial gasification step. Both of the stages were carried out in a conventional tubular furnace at a heating rate of 10 °C/min. until reaching the desired temperature. Carbonization was carried out at 800 °C for 2 h under continuous nitrogen flow (150 cm^3^/min. STP). Afterwards, the carbonized precursor was activated at the same temperature in a CO_2_ flow (150 cm^3^/min. STP) for 7 h, reaching a 45% burn-off. The final activated carbon that was obtained by this activation process presented a yield of 13.7% based on dried olive stone and it was denoted as AC800.

### 2.2. Zr Loading over the Different Activated Carbons

Zr loading was carried out over the different activated carbons by pore-volume impregnation of the dried samples. An appropriate amount of ZrO(NO_3_)_2_ solution was added to a known quantity of activated carbon to prepare the catalysts with a Zr load ranging from 0.75 to 7.5% (*w/w*). The impregnated carbons were dried overnight at 120 °C and finally calcined at 250 °C for 2 h in air. Adding –ZrX to the name of the parent activated carbon denoted the catalysts that were obtained, where X refers to the Zr loading mass percentage (e.g., ACP2800Zr7.5). 

### 2.3. Characterization of Carbon Catalysts

The porous texture of the prepared activated carbons and catalysts was characterized by N_2_ adsorption–desorption at −196 °C and by CO_2_ adsorption at 0 °C, carried out in an ASAP 2020 equipment (ASAP 2020, Micromeritics Instruments Corp., Norcross, GA, USA). The samples were previously outgassed for at least 8 h at 150 °C. From the N_2_ adsorption-desorption isotherm, the apparent surface area (A_BET_) was determined by applying the BET equation [39]. The α_s_ method was used to obtain the values of the so-called external surface area (A_s_), that is, the surface area associated to the non-microporous structure; and, the micropore volume (V_s_), while using the high-resolution method that was proposed by Kaneko et al. [40] with a nonporous carbon black sample (Elftex-120) as standard [41]. The narrow mesopore volume was determined as the difference between the adsorbed volume of N_2_ at a relative pressure of 0.99 and the micropore volume, V_s_. From the CO_2_ adsorption data, the narrow micropore volume (V_DR_) and surface area (A_DR_) were calculated while using the Dubinin–Radushkevich equation [42].

The surface chemistry of the samples was analyzed by X-ray photoelectron spectroscopy (XPS) and temperature programmed desorption (TPD). XPS analyses of the samples were obtained by a VersaProbe II ESCA 5701 model Physical Electronics apparatus (VersaProbe II ESCA 5701, Physical Electronics, Chanhassen, MN, USA), with Al Kα radiation (1486.6 eV). For the analysis of the XPS peaks, the maximum of the C1s peak was set at 284.5 eV and used as a reference for the other peaks [35]. Temperature programmed desorption (TPD) provides information regarding the nature of the oxygen groups present on the surface of activated carbons [43]. The TPD profiles were obtained in a custom fixed-bed reactor (Forns Hobersal, Barcelona, Spain) that was placed inside an electrical furnace. The samples were heated from room temperature to 1600 °C at a heating rate of 10 °C/min. in a N_2_ flow (200 cm^3^ STP/min). The amounts of CO and CO_2_ desorbed from the samples were monitored by nondispersive infrared (NDIR) gas analyzers (ULTRAMAT 22, Siemens AG, Munich, Germany).

The total acidity and acid strength distribution of the prepared catalysts was determined by temperature programmed desorption of ammonia (NH_3_-TPD). The NH_3_-TPD was carried out while using 100 mg of dried material, which was saturated with NH_3_ (20% (*v/v*) in helium) for 15 min at 100 °C. After saturation, the weakly adsorbed NH_3_ was desorbed in a helium flow at 100 °C until no NH_3_ was detected in the outlet stream. The NH_3_-TPD was performed by raising the temperature up to 550 °C at a heating rate of 10 °C/min. Mass spectroscopy monitored the outlet NH_3_ concentration (Pfeiffer Omnistar GSD-301, Pfeiffer Vacuum Technology AG, Asslar, Germany), registering the signal *m/z* 17.

### 2.4. Methanol Dehydration Catalytic Reaction

The carbon materials that were prepared were used as catalysts in the dehydration of methanol in the gas phase at atmospheric pressure in a fixed bed microreactor (i.d. 4 mm) (MERVILAB, Madrid, Spain) that was placed inside a vertical furnace (TR2-A, Forns Hobersal, Barcelona, Spain) with temperature control. In a typical experiment, 200 mg of catalyst (100–300 μm particle size) were used. Methanol was fed to the system by using a syringe pump (Cole-Parmer^®^ 74900-00-05 model, Cole-Parmer Instrument Company, Vernon Hills, IL, USA), ensuring a constant controlled methanol flow, 0.37 g/h. The reaction was carried out in a helium atmosphere in a temperature range of 250–450 °C. All of the lines were heated up to 120 °C to avoid condensation of any compound. The feed conditions that were used were methanol partial pressures of 0.02 and 0.04 atm, water vapor partial pressures of 0 and 0.02 atm and a space time of 0.1 (g·s/μmol_MeOH_). The outlet gas concentrations were quantified by on-line gas chromatography (Perkin-Elmer Clarus 500 GC that was equipped with TCD (thermal conductivity detector) and FID (flame ionization detector) detectors (Clarus 500 GC, Perkin-Elmer, Waltham, MA, USA). The used columns were a Permanent gases active carbon 80/100 mesh for CO and CO_2_ analysis (Teknokroma Analítica, Barcelona, Spain) and a 1.9 m × 1/8″ × 2.1 mm Porapak N 80/100 + 0.5 m × 1/8″ × 2.1 mm Porapak Q 80/100 column (Teknokroma Analítica, Barcelona, Spain) for methanol, DME, and light hydrocarbons separation. 

The conversion was defined as the ratio of the amount of methanol converted to the amount of methanol that is supplied to the reactor. The selectivity (in mol%) was defined as the ratio of carbon moles in a specific product divided by the moles of converted methanol. The carbon balance was reached with an error lower than 3% in all of the experiments.

## 3. Results and Discussion

### 3.1. Characterization of the Carbon Catalysts 

**Figure 1:**
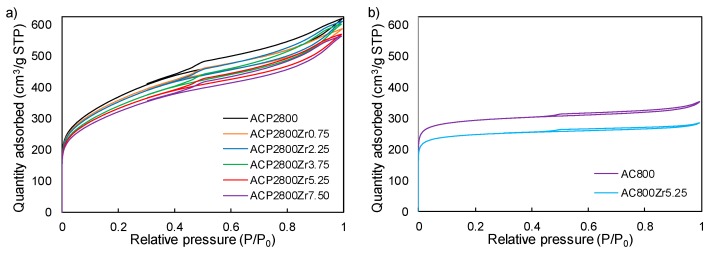
Nitrogen adsorption–desorption isotherms at −196 °C of (**a**) Chemically activated carbon (ACP2800) before and after different Zr loads. (**b**) Physically activated carbon (AC800) before and after a Zr load of 5.25% (*w/w*).

Figure 1 shows the N_2_ adsorption-desorption isotherms at −196 °C for the different carbon materials that were prepared in this study. Chemically activated carbon, ACP2800, (Figure 1a) presents a type I b isotherm [44] corresponding to materials that have a broad range of wider micropores and narrow mesopores. The exhibition of an open knee is noticeable, which extends up to increasingly higher relative pressures and it is characteristic of a wide microporosity and mesoporosity. An H4 type hysteresis loop is observed, closing at a relative pressure value of 0.4, associated to capillary condensation in mesopores. Physically activated carbon, AC800, (Figure 1b) shows a type I a isotherm, which is characteristic of a typical microporous solid, adsorbing almost all N_2_ volume at very low relative pressures.

The results show that the porosity of the sample ACP2800 (Figure 1a) seems not to be highly affected by the Zr loading. In this sense, a slight decrease in the volume of N_2_ adsorbed at low relative pressures is only observed, being associated to a reduction of the wide microporosity, due to Zr deposition. The physically activated carbon, AC800 (Figure 1b), also experienced a porosity decrease after Zr loading, given that a reduction in the volume of N_2_ was also observed, but in this case at very low relative pressures, which are associated to narrow micropore reduction or blockage.

Table 1 presents the values of the textural parameters, which were calculated from N_2_ adsorption-desorption and CO_2_ adsorption isotherms, of the prepared activated carbons. ACP2800 presents a value of A_BET_^N2^ higher than that of the A_DR_^CO2^, which is indicative of a wide porous texture in this sample. The higher value of mesopore volume (V_mes_) is also noticeable when comparing with the micropore value (V_s_), indicating a high contribution to mesoporosity. The external surface area value (A_s_) confirms this fact. Physically activated carbon (AC800) presents a specific surface area that is similar to the one that was obtained for the chemically activated carbon (ACP2800). However, the physically activated carbon (AC800) presents an A_BET_^N2^ value that was comparable to the A_DR_^CO2^ value, which is characteristic of a typical microporous solid. Moreover, the similarity between the values of V_s_^N2^ and V_DR_^CO2^ evidences the presence of a narrow microporous structure. The results show that, after Zr loading, all of the samples experiment a reduction in the structural parameters. On the one hand, a decrease in the V_s_^N2^ values is observed when Zr loading is increased over chemically activated carbons, ACP2800ZrX. However, V_DR_^CO2^ ones seem to be not highly affected, when increasing the Zr loading, which is indicative of Zr deposition on the surface of the wider micropores. On the other hand, when the physically activated carbon, AC800, is loaded with Zr, V_s_^N2^, and V_DR_^CO2^ values show a similar decrease, indicating that Zr deposition is blocking the narrow microporosity.

X-ray photoelectron spectroscopy (XPS) analyzes were carried out in order to evaluate the surface element distribution and the surface chemical composition of the samples. Both of the activated carbons, ACP2800 and AC800, are mainly composed of carbon (89.5 and 95.4% (*w/w*), respectively) and oxygen (7.2 and 4.6% (*w/w*), respectively). However, chemically activated carbon also presents a low, but still significant, surface concentration of phosphorus (3.2% (*w/w*)), which has been attributed to the activation step [26,32]. Chemical activation of olive stone (and other biomass waste) with phosphoric acid seems to proceed by forming phosphate and polyphosphate bridges that crosslink the biopolymer fragments, developing the porous texture and generating these phosphorus complexes that remain chemically stable bonded to the carbon surface, even despite the washing step [34]. The P concentration for this sample was 3.6% (*w/w*), when it was analyzed by ICP-MS (Inductively coupled plasma mass spectrometry). The similar value that was obtained by both analytical techniques suggests that the phosphorus surface groups are very well distributed on the carbon particle surface. 

Table 2 presents the atomic surface ratios of Zr, O, and P with respect to each other, as calculated for the different Zr loading samples. As expected, the Zr/P ratio increases and the O/Zr ratio decreases, as the zirconium amount augments on the samples. The O/P ratio should linearly increase as the zirconium loading increases, since the salt used for the impregnation, ZrO(NO_3_)_2_, decomposes yielding ZrO_2_. However, it seems that at low zirconium loadings, this ratio is not highly affected. This fact could indicate that zirconium tends to bond oxygen species present on the carbon surface at low metal loadings, such as C-O-PO_3_ and C-O, hence less oxygen being necessary for the coordination of the zirconium atoms. Nevertheless, it seems that the zirconium that is incorporated to the carbon surface is accompanied by oxygen atoms at high Zr loadings, thus forming zirconium oxide species on the carbon surface. 

**Figure 2:**
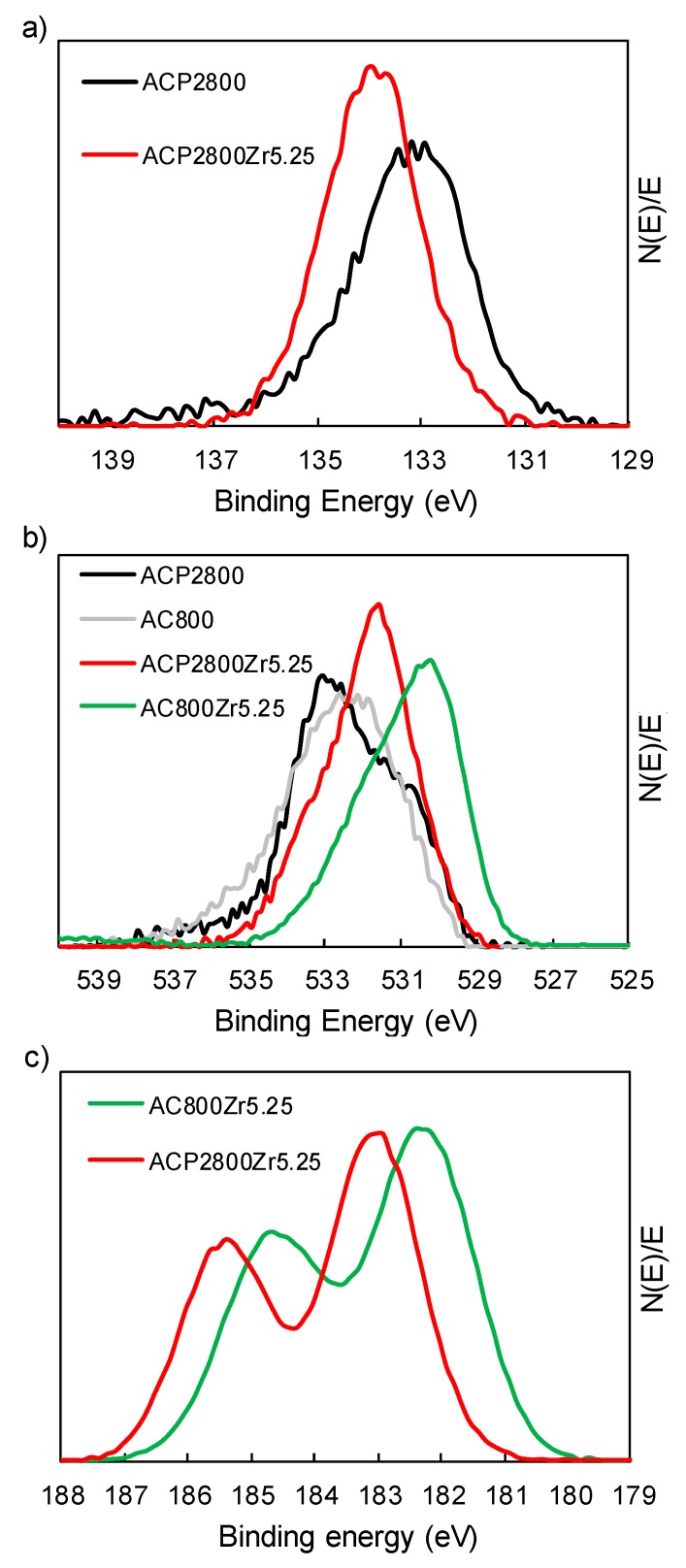
(**a**) Normalized P2p XPS spectra for ACP2800 and the 5.25% Zr loaded phosphorus containing samples; (**b**) Normalized O1s XPS spectra for ACP2800, AC800 and the 5.25% Zr loaded samples; and, (**c**) Normalized Zr3d XPS spectra for the 5.25% Zr loaded samples.

The XPS spectra were examined in order to analyze the chemical surface of the Zr-loaded samples in depth. Figure 2 shows the normalized high resolution XPS spectra for phosphorus, oxygen, and zirconium. XPS deconvolutions can be found in the supplementary information (Figures S1–S3). The P2p spectrum of sample ACP2800 shows a main peak at a binding energy around 133 eV, which is characteristic of pentavalent tetracoordinated phosphorus [35,45]. The normalized O1s spectra for both chemically and physically prepared activated carbons show a band with a maximum at a binding energy of 532.3 eV, which is attributed to the single bonded oxygen, in the form of C-OH, C-O-C, and/or C-O-P. It can be observed that the former shows a more pronounced shoulder at a binding energy of 530.7 eV when comparing the O1s spectrum for the chemically activated carbon with the spectrum for the physically activated one, which is characteristic of C=O and/or P=O groups. 

After Zr loading, all of the prepared phosphorus-containing activated carbons present similar XPS spectra. Due to this similarity, only the spectra that were obtained for sample ACP2800Zr5.25 have been shown. The normalized P2p spectrum that was obtained for this sample (Figure 2a) shifts to higher binding energy values when it is compared to its parent carbon, ACP2800 (see Figure S1). This displacement is a consequence of the oxidation of the phosphorus groups and now the peak appears at the binding energy characteristic of phosphates and/or polyphosphates, at 133.7 eV [34,35]. It can also be observed that the O1s and Zr3d normalized spectra for the carbons containing and not containing phosphorus exhibit significant differences between them. The normalized O1s band that was obtained for sample AC800Zr5.25 (not containing phosphorus) consists of a main peak at a binding energy of 530.2 eV, which is assigned to oxygen in metal oxides [46] and a small and broad high binding energy shoulder, which could be attributed to surface hydroxyl species, such as Zr-OH and/or C-OH [46,47]. In contrast, the normalized O1s band that was obtained from the phosphorus-containing sample, ACP2800Zr5.25, can be deconvoluted in three peaks (see Figure S2). The main peak, which appears in this case at a binding energy of 532 eV, is associated to the oxygens of the phosphate groups. Two shoulders are also observed, one at higher binding energies that corresponds to the acidic P-OH groups and the other one at lower binding energies, which is probably due to the Zr-OH groups [48]. The normalized Zr3d band exhibits the two 3d peaks that are separated by 2.3 eV, which are characteristic of the tetravalent Zr^4+^, in both cases (see Figure S3). However, it is noteworthy that the binding energy at which the Zr3d appears is different when phosphorus is present in the sample. In this sense, it has been reported in the literature, when analyzing zirconium phosphates [45,48], that the coordination of Zr atoms with a high number of strongly polarized oxygens results in the displacement of the Zr3d band to higher binding energies [48]. The presence of these species could be responsible for the higher binding energy that was found in the spectra of the phosphorus-containing samples (see ACP2800Zr5.25 in Figure 2c). 

**Figure 3:**
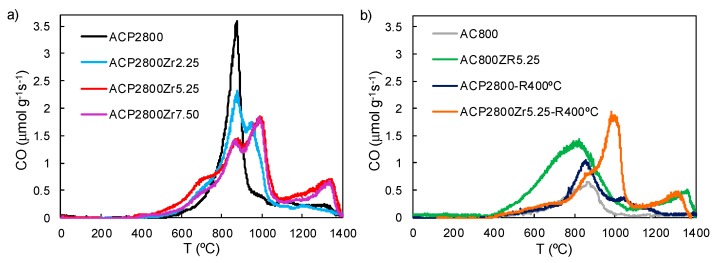
Amount of CO that evolved as a function of temperature during temperature programmed desorption (TPD) for (**a**) ACP2800, ACP2800Zr2.25, ACP2800Zr5.25 and ACP2800Zr7.50. (**b**) AC800, AC800Zr5.25 and, ACP2800 and ACP2800Zr5.25 after 4 hours on reaction at 400 °C, P_MeOH_ = 0.02 atm, W/F_MeOH_ = 0.1 g.s/μmol.

The TPD technique also analyzed the surface chemistry of the samples, which is used to characterize the oxygen groups on the surface of carbons in detail. Figure 3a shows the evolution of CO from the TPD analysis of the phosphorus-containing sample, ACP2800, before and after the different Zr loadings. ACP2800 releases most of the CO at a temperature around 870 °C, which has been previously assigned to the decomposition of stable C-O-P type bonds of C-O-PO_3_ surface groups, producing CO gas and C’-P type bonds of C-PO_3_ surface groups, where C’ represents a new carbon center [26,49]. After Zr loading on ACP2800, the CO profiles show three main peaks at 870, 1000, and 1300 °C, respectively. As it can be observed, the peak at 870 °C, which was associated to the presence of C-O-P bonds, decreased as the amount of Zr that was deposited on the carbon surface increased. On the other hand, the amount of CO that evolved at 1000 and 1300 °C increased with metal loading and it was related to the presence of zirconium species on the carbon surface. Moreover, it has to be noted that the total amount of CO evolved in the temperature range of 750–1100 °C was the same for all the samples, with a linear relationship being observed between the decrease in CO evolving at 870 °C and the increase in CO evolving at 1000 °C. The TPD CO profiles obtained for the samples not containing phosphorus, AC800 and AC800Zr5.25, were also studied in order to thoroughly analyze the nature of these surface complexes that evolved as CO at such a high temperature (see Figure 3b). AC800 presented a CO evolution peak at about 900 °C, which was associated to carbonyl/quinone or ether groups [34]. After Zr loading, the presence of a second peak at a temperature of 1300 °C was noticeable (see AC800Zr5.25 in Figure 3b), which has been related to the presence of C-O-Zr bonds and to the surface ZrO_2_ carboreduction [50]. However, the CO peak that appeared at about 1000 °C was not present in this sample. Therefore, the CO that evolved at this temperature must be related to the simultaneous presence of zirconium and phosphorus, mainly in the form of zirconium phosphate groups (as already revealed by the XPS analysis), bonded to the carbon surface. Furthermore, the relationship that was observed between the decreased amount of CO that evolved at 870 and the increased amount evolved at 1000 °C with increasing Zr loading seems to indicate that the C-O-P type bonds were cleaved in favor of the formation of zirconium phosphate species. These zirconium phosphate species seem to be bonded to the carbon surface via C-O-P and C-O-Zr bonds, forming C-O-(P-O-Zr)-O-C like structures, which decompose, releasing CO at 870 °C and 1000 °C, respectively. According to these TPD results, the number for C-O-Zr bonds of the zirconium phosphate surface groups seems to be higher than the one for C-O-P bonds in the samples with a Zr loading that was larger than 2.25. 

The CO that evolved at temperatures below 800 °C for all of the samples that were loaded with Zr can be attributed to the presence of anhydride, phenol, and/or ether surface groups [43]. It is worth noting that the CO amount that evolved below this temperature was almost the same for all of the phosphorus-containing carbon samples loaded with Zr (see Figure 3), and therefore did not seem to have any relationship with the amount of metal loaded. These oxygenated species were probably formed during the calcination stage, at 250 °C [34]. The CO evolution at temperatures below 800 °C in the AC800Zr5.25 sample was higher than the one that was observed in the phosphorus-containing samples, due to the lower oxidation resistance of the non-phosphorus-containing activated carbons as compared to the ones containing phosphorus [31].

**Figure 4:**
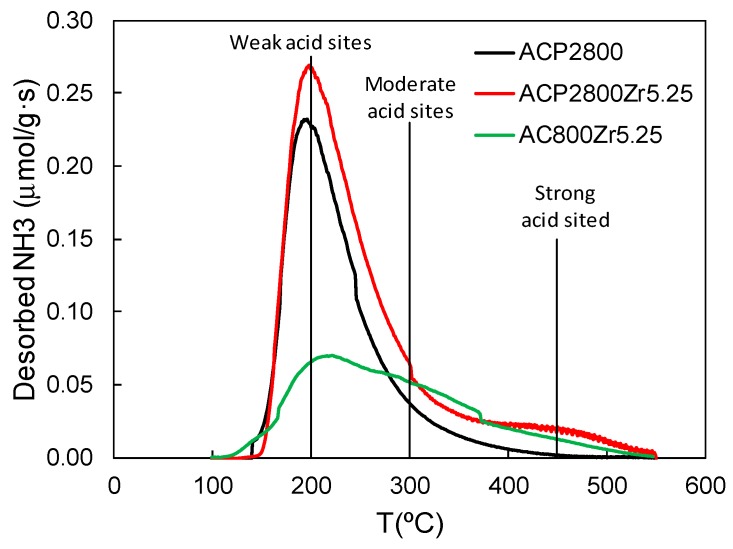
Ammonia TPD of the samples ACP2800, ACP2800Zr5.25, and AC2800Zr5.25.

Figure 4 presents the NH_3_-TPD profiles that were obtained for ACP2800, ACP2800Zr5.25, and AC800Zr5.25. The temperature at which ammonia desorption took place indicates the acid strength of the catalytic sites, with the sites desorbing at a lower temperature being weaker. ACP2800 desorbed most of the ammonia at a temperature of 200 °C, which is associated to weakly (or moderately) adsorbed ammonia on the C-O-P groups, which are present on the surface of this material. The presence of a shoulder in the curve at a temperature of 250 °C and a large tail at higher temperatures is also noteworthy, being associated to the stronger adsorption of NH_3_ on the P-OH groups present on the activated carbon surface, which act as Brönsted acid sites [36,51]. ACP2800Zr5.25 showed a similar profile to its parent carbon (ACP2800). However, it exhibited a slightly higher total amount of desorbed ammonia (175 μmol/g for ACP2800Zr5.25 vs 128 μmol/g for ACP2800). This increase in the acidity could be associated to the presence of Zr-O-P groups, which can act as acid sites. AC800 did not desorb any appreciable amount of ammonia during the NH_3_-TPD analysis in contrast with the chemically activated carbon, which suggests that this carbon lacks the presence of acid surface groups. Once this carbon was loaded with Zr in the same conditions as those for the preparation of ACP2800Zr5.25, the resulting carbon, AC800Zr5.25, which did not contain phosphorus, exhibited a much lower amount of desorbed ammonia, 81 μmol/g, during the NH_3_-TPD analysis than the one that was observed in the phosphorus-containing sample. The acidity of this sample (AC800Zr5.25) can be attributed to the presence of zirconium on the carbon surface, forming zirconium oxide.

### 3.2. Catalytic Dehydration of Methanol 

Methanol dehydration was studied for the carbon-based catalysts in a fixed bed reactor. In the absence of catalysts, no reaction occurred below 600 °C. Heat and the mass transfer limitations were theoretically checked and considered to be negligible according to the criterion that was suggested by Moulijn et al. [52].

#### 3.2.1. Effect of Zr Loading

**Figure 5:**
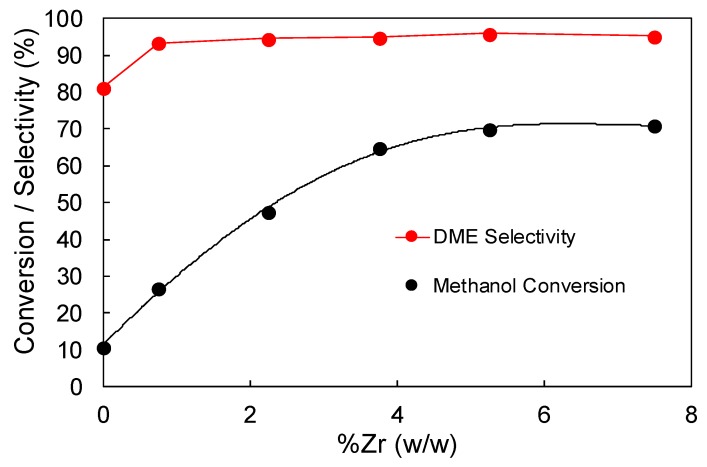
Steady-state methanol conversion and selectivity to dimethyl ether (DME) as a function of Zr loading for the sample ACP2800 (400 °C, P_MeOH_ = 0.02 atm, W/F_MeOH_ = 0.1 g.s/μmol).

Figure 5 shows the steady-state methanol conversion and selectivity towards DME that was obtained for the phosphorus-containing activated carbon, ACP2800, loaded with different amounts of Zr (ACP2800ZrX), at a reaction temperature of 400 °C (P_MeOH_ = 0.02, W/F_MeOH_ = 0.1 g.s/μmol). At low metal loadings, the steady state methanol conversion increases almost linearly with the amount of zirconium being added to the P-containing activated carbon. However, it seems that there is a value of Zr loading (around 5%) from which no further increase in the methanol conversion is observed, with the methanol conversion remaining constant at 69%. The selectivity towards DME does not show a dependence with the metal loading, always presenting a value that is higher than 95%. According to these results, it seems that a Zr loading of 5.25% could be considered to be the optimum one, in terms of methanol conversion, among the prepared catalysts. 

#### 3.2.2. Effect of Temperature

**Figure 6:**
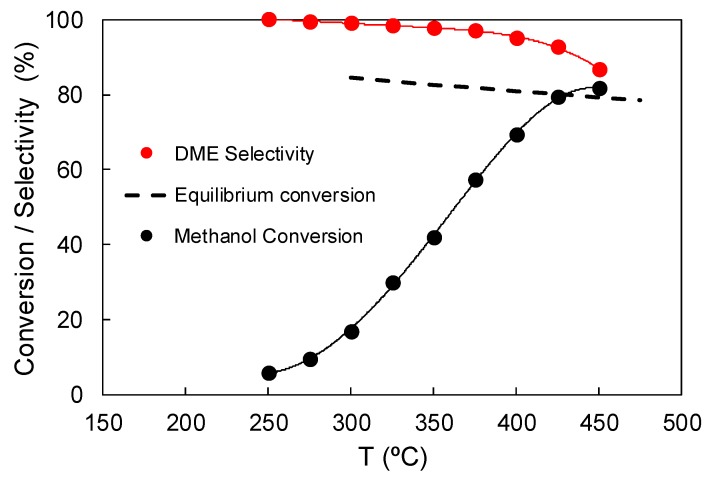
Methanol conversion and selectivity to DME as a function of the temperature for the catalyst ACP2800ZR5.25 (P_MeOH_ = 0.02 atm, W/F_MeOH_ = 0.1 g.s/μmol).

Figure 6 represents the methanol steady-state conversion and the selectivity towards DME as a function of the reaction temperature for the sample ACP2800Zr5.25. As expected, an increase in the reaction temperature produces an increase in the methanol steady state conversion. Regarding the selectivity towards DME, the high values exhibited by this carbon catalyst (higher than 95%) have to be pointed out, even at temperatures as high as 400 °C. The selectivity towards DME begins to decrease at temperatures that are higher than 425 °C and CH_4_ and CO appears as the main by-products, resulting from methanol/DME decomposition.

#### 3.2.3. Effect of Phosphorus on the Carbon Support

**Figure 7:**
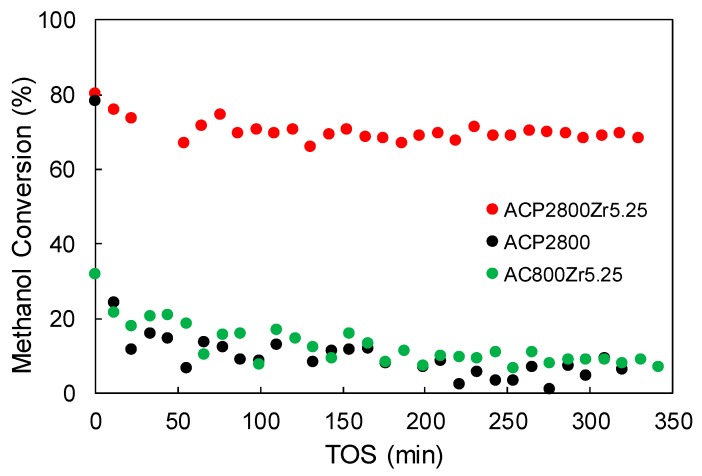
Methanol conversion as a function of time-on-stream (TOS) for ACP2800, ACP2800Zr5.25m and AC800Zr5.25 (400 °C, P_MeOH_ = 0.02 atm, W/F_MeOH_ = 0.1 g.s/μmol).

Figure 7 shows the methanol conversion, X_MeOH_, as a function of time-on-stream (TOS) for ACP2800, ACP2800Zr5.25, and AC800Zr5.25 at the reaction temperature of 400 °C (P_MeOH_ = 0.02 atm, W/F_MeOH_ = 0.1 g.s/μmol). Physically activated carbon (AC800), which did not contain P surface groups, did not show any catalytic activity. After Zr loading, AC800Zr5.25 showed some activity (an initial methanol conversion of 30%), but this decreased with time on stream, evidencing a fast deactivation process. Chemically activated carbon (ACP2800), which did contain P surface groups, exhibited a high initial methanol conversion (of about 80%). However, this value dropped in the first minutes of reaction, which indicated that the catalyst suffered from fast deactivation. Nevertheless, this P-containing carbon material that was loaded with Zr (ACP2800Zr5.25 catalyst) achieved steady-state conditions after a short period of time, showing a constant methanol conversion of 69% and a selectivity to DME that was higher than 95%, as already shown in Figure 6. 

In a previous work, it was highlighted that ACP2800 presented P-OH and C-O-P type acidic surface sites, as in C-O-PO_3_ surface groups [34]. It was also reported that the P-OH and C-O-P type sites acted as strong Brönsted and weak and/or moderate acid sites, respectively, for the methanol dehydration, although both of them were deactivated by coke deposition if oxygen was not present in the reaction gas mixture [37]. In this sense, when analyzing the CO profile that was obtained from TPD experiments for this sample after reaction (see ACP2800-R400 °C in Figure 3b), a decrease of the CO that evolved at 870 °C can be observed, which is associated to a lower presence of C-O-P groups on the carbons surface after the reaction. Moreover, XPS analyses showed an increase of 5% in the carbon content for this sample, evidencing the coke deposition on the P and O surface species. 

In the case of AC800Zr5.25, the Zr loading only resulted in the formation of zirconium oxide species on the surface of the material (due to the absence of phosphorus in its surface), as revealed by XPS. This species provided the carbon material a low surface acidity and, thus, a low catalytic activity. Moreover, the low selectivity towards DME (34%) evidenced that methanol decomposition, rather than methanol dehydration, was taking place in this case, as it has been reported with electrospun zirconia nanofibers [53].

ACP2800Zr5.25 mainly presented zirconium phosphate species on its surface, as revealed by XPS. C-O-P type surface sites that are present on this catalyst seemed to be deactivated by coke formation, as was revealed by the methanol conversion decay that this sample exhibited before reaching the steady state conversion, as occurred for the sample ACP2800. In this case, the CO profile that was obtained from TPD analyses for this sample after reaction (see ACP2800Zr5.25-R400 °C in Figure 3b) showed that the peak at 870 °C decreased when compared to the one for the fresh sample, indicating the lower presence of C-O-P surface sites. However, the peak at 1000 °C remained unaltered after reaction, indicating that the C-O-Zr bonds forming the zirconium phosphate structure are unaltered during the reaction. The steady-state methanol conversion that was achieved by this catalyst can be associated to the presence of zirconium phosphate species that are bonded to the carbon surface. 

The existence of an optimum Zr loading at about 5% (*w/w*) (see Figure 5), in terms of catalytic activity, can be also explained according to the fact that zirconium phosphate groups are the actives species that are involved in the methanol dehydration reaction, in this case. The amount of phosphorus that is present on the carbon surface of ACP2800 catalyst is limited by the activation process [35,36]. In this sense, the amount of Zr is limiting the zirconium phosphate formation at low metal loadings, so the number of active sites is directly proportional to the metal loading. On the opposite, at high metal loadings, the amount of surface phosphorus is limiting the zirconium phosphate species formation and the extra amount of Zr added is forming zirconium oxide species, which does not produce significant improvement in the steady state methanol conversion, as it was observed for Zr loading higher than 5% (*w/w*) (see Figure 5). 

**Figure 8:**
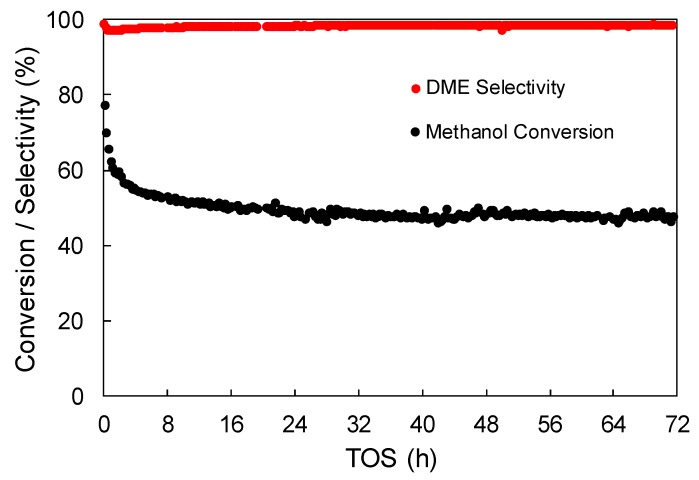
Methanol conversion and selectivity towards DME as a function of TOS for the ACP2800Zr5.25 sample (350 °C, P_MeOH_ = 0.04 atm, W/F_MeOH_ = 0.1 g.s/μmol).

A long-term experiment was carried out with the aim of studying the stability of the ACP2800Zr5.25 catalyst (350 °C, P_MeOH_ = 0.04 atm, W/F_MeOH_ = 0.1 g.s/μmol). Figure 8 depicts the evolution of methanol conversion and selectivity towards DME as a function of time-on-stream (TOS). Once the catalyst achieved the steady-state methanol conversion, the catalytic activity remained unaltered for more than 72 h, as it can be observed. The selectivity towards DME also remained higher than 97%, without being affected by TOS. 

#### 3.2.4. Effect of Water Vapor in the Feed

The industrial methanol synthesis process, as well as DME production, involves the generation of considerable amounts of water vapor. It has been reported that the presence of water affects the catalytic activity and stability of the methanol dehydration catalysts [2,54]. In this sense, Akarmazyan et al. [15] reported a decrease in the methanol conversion, being higher than 20%, after five hours on stream when a 10% of water vapor was present in the reactor feed, while using Al_2_O_3_ as catalyst. This behavior was associated to the competitive water adsorption on the active sites [55]. 

**Figure 9:**
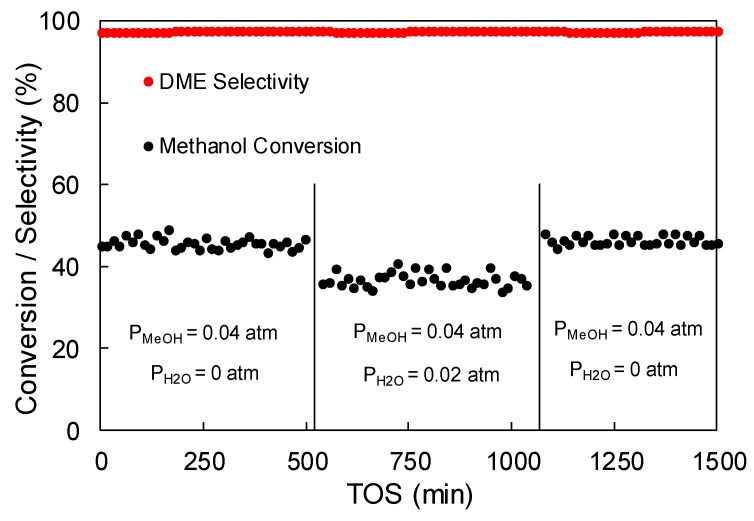
Effect of water vapor in the feed on methanol conversion at 350 °C (W/F_MeOH_ = 0.1 g.s/μmol).

The influence of water vapor in the reaction gas mixture on the catalytic dehydration of methanol was studied for ACP2800Zr5.25 in order to address this issue. Figure 9 shows the evolution of methanol conversion with time-on-stream in the presence and absence of water vapor in the reactor feed. As it can be observed, only a slight decrease, lower than 10%, in the initial methanol conversion is observed for this catalyst. However, it is important to mention that the methanol conversion did not change with time-on-stream at a constant water vapor partial pressure, unlike the reports for other catalysts [54]. Moreover, the methanol conversion was restored to the value that was obtained in the absence of water after removing the water vapor from the feed, evidencing that the effect of water on the catalytic performance is reversible. This behavior was also observed for other water vapor pressures studied. It is also remarkable that the presence of water vapor did not affect the selectivity towards DME, a selectivity value higher than 95% was always observed for all of the water vapor pressures studied.

In the light of all the catalytic results that are presented in this study, it is remarkable that the catalyst here presented could be used in the whole range of temperatures used in the industrial methanol dehydration process (250–400 °C). Catalysts for the selective methanol dehydration to DME must show enough activity for this reaction. However, too high activity resulted in the production of hydrocarbons. Zeolites have been widely investigated [56,57,58]. In this sense, Rutkowska et al. [58] analyzed the catalytic performance of different zeolitic materials, pointing out a high activity when using these materials. However, they also claimed that, at temperatures higher than 275 °C, the selectivity to DME dropped as a result of hydrocarbons formation, in the most active catalysts. Moreover, they also reported that the stability of these materials also depended on the reaction temperature, with these catalysts becoming deactivated at temperatures above 275 °C due to coke formation. Similar results have been reported for other catalysts. Nitta et al. [59] studied different sulfated zirconia catalysts for methanol dehydration, reporting a low selectivity to DME at 350 °C, due to hydrocarbons formation. Cheng et al [18] also reported the formation of hydrocarbons at 350 °C when using pillared zirconium phosphates as catalysts. Alharbi et al. [19] studied the use of different tungsten Keggin heteropolyacids as a catalyst for this reaction, exhibiting, these materials, activities higher than zeolites. Nevertheless, the formation of hydrocarbons started occurring at temperatures above 200 °C, decreasing the DME selectivity. Moreover, as it was observed for zeolites, this hydrocarbon production was accompanied by a catalyst deactivation due to coke formation. 

When comparing the catalysts that are presented in this study with other catalysts that are reported in the literature, it has first to be noted that this material counts on the noticeable advantage of having been prepared from an inexpensive agri-food industrial waste. Regarding the activity, it is important to mention that, in contrast to other inorganic acid solids, such as alumina, zeolites, and Keggin heteropoly acids, in which the whole material is considered as active phase, only 5% (*w/w*) of this waste-derived material (for the best catalyst presented in this study) can be considered as active phase. Moreover, the operating conditions that were used in this study are significantly less favorable for achieving high conversion values. In this sense, space velocities up to 100 times higher than other studies [19] and considerably lower methanol partial pressures [60]. Despite the conditions that were used in this study, the material presented herein showed remarkable activity. In addition, when comparing the selectivity to DME and the stability on stream, this biomass-derived material exhibits noticeable features. In this regard, it has been shown that the catalyst presented here exhibits a selectivity to DME higher than 95% in the whole range of temperature studied (250–400 °C). Furthermore, this waste-derived catalyst shows a high chemical and hydrothermal stability under the operation conditions that were studied.

## 4. Conclusions

Two kinds of activated carbons that were prepared by chemical (with phosphoric acid) and physical (by CO_2_ partial gasification) activation of olive stone, an agri-food industry waste, were loaded with different Zr amounts and used as catalysts for the selective dehydration of methanol to dimethyl ether.

The presence of chemically stable phosphorus surface groups, mainly in the form of C-O-PO_3_ groups, on the activated carbon prepared via chemical activation with phosphoric acid, seems to have great relevance in the catalytic performance of these carbon materials for methanol dehydration. XPS analysis revealed that zirconium bonded these phosphorus complexes during the metal loading, resulting in the formation of zirconium phosphate species on the activated carbon surface. 

Phosphorus-containing activated carbon, ACP2800, presented a high initial methanol conversion. However, the conversion dropped in the first minutes of the reaction, evidencing a deactivation process. Once loaded with zirconium, the resulting material presented a relatively high catalytic activity and steady-state methanol conversion was observed, without deactivation. The catalytic performance was improved with the amount of zirconium that was added to the activated carbon until a fixed loading amount (of around 5% (*w/w*)), from which no further increasing in the steady-state methanol conversion was observed. The catalytic activity of these materials was attributed to the presence of well dispersed surface zirconium phosphate species, whose formation was limited by the amount of phosphorus that was present on the carbon surface of the chemically activated carbon.

Zr-loaded phosphorus-containing activated carbons exhibited methanol conversion values that were close to thermodynamic equilibrium, keeping selectivity towards DME higher than 95% in the whole range of temperatures that were used in the industrial DME synthesis (250–400 °C). The stability results showed that, once the materials achieved the steady-state methanol conversion, it remained unaltered for more than 72 h.

The presence of water vapor in the reaction gas on the catalytic dehydration of methanol was studied. The results showed that the methanol conversion decreased when increasing the water vapor partial pressure. However, the methanol conversion was not affected with time-on-stream at a constant water vapor partial pressure. Moreover, the methanol conversion was restored to the value that was obtained in the absence of water once removing the water vapor from the feed, evidencing that the effect of water on the catalytic performance was reversible. The selectivity towards DME was not affected by the presence of water vapor in the feed, with a value higher than 95% being observed for all of the water vapor pressures studied.

## Figures and Tables

**Table 1:** Characteristic parameters of the porous texture and zirconium loading for the prepared activated carbons.

Sample.	N_2_ Isotherm					CO_2_ Isotherm		%Zr
	A_BET_^N2 (a)^ (m^2^/g)	V_p_^N2 (b)^ (cm^3^/g)	V_mes_^N2 (c)^ (cm^3^/g)	As^N2 (d)^ (m^2^/g)	Vs^N2 (d)^ (cm^3^/g)	A_DR_^CO2 (e)^ (m^2^/g)	V_DR_^CO2 (e)^ (cm^3^/g)	
ACP2800	1280	0.95	0.44	310	0.52	471	0.19	0
ACP2800Zr0.75	1273	0.90	0.41	308	0.49	519	0.21	0.75
ACP2800Zr2.25	1195	0.94	0.45	297	0.50	511	0.21	2.25
ACP2800Zr3.75	1148	0.93	0.44	281	0.49	501	0.20	3.75
ACP2800Zr5.25	1143	0.88	0.40	247	0.48	497	0.20	5.25
ACP2800Zr7.5	1132	0.86	0.40	242	0.47	487	0.20	7.50
AC800	1127	0.55	0.10	52	0.44	865	0.35	0
AC800Zr5.25	812	0.44	0.07	43	0.37	757	0.30	5.25

a: Calculated from BET equation. b: Obtained from N_2_ adsorption isotherm at P/P_0_ = 0.95.c: Obtained by difference between Vp and Vs. d: Calculated from N_2_ adsorption isotherm by using the αs method. e: Calculated from CO_2_ adsorption isotherm by using the Dubinin–Radushkevich equation.

**Table 2:** Atomic surface ratios of Zr, O, and P with respect to each other, obtained by X-ray photoelectron spectroscopy (XPS) analysis, for the Zr-loaded P-containing activated carbons.

Sample	Atomic Surface Ratios (XPS)		
	Zr/P	O/Zr	O/P
ACP2800	-	-	4.45
ACP2800Zr0.75	0.49	9.75	4.70
ACP2800Zr2.25	0.50	9.06	4.57
ACP2800Zr3.75	0.62	8.98	5.55
ACP2800Zr5.25	0.89	7.52	6.71
ACP2800Zr7.50	0.98	7.16	7.00

## Supplementary Materials

The following are available online at https://www.mdpi.com/1996-1944/12/13/2204/s1, Figure S1: Deconvolution of the normalized P2p XPS spectra for ACP2800 and the 5.25% Zr loaded phosphorus containing sample. Figure S2: Deconvolution of the normalized O1s XPS spectra for ACP2800, AC800 and the 5.25% Zr loaded samples (ACP2800Zr5.25 and ACG800Zr5.25). Figure S3: Deconvolution of the normalized Zr3d XPS spectra for the 5.25% Zr loaded samples, ACP2800Zr5.25 and ACG800Zr5.25.
