# Supplementary material for: Methanol Dehydration to Dimethyl Ether on Zr-Loaded P-Containing Mesoporous Activated Carbon Catalysts
Source: Materials (Basel). 2019 Jul 9;12(13):2204. doi: 10.3390/ma12132204 (PMC6651118; doi:10.3390/ma12132204)
Supplement: Supplementary file 1 [file materials-12-02204-s001.pdf]

materials

MDPI

Article

Methanol Dehydration to Dimethyl Ether on Zr-Loaded P-Containing Mesoporous Activated Carbon Catalysts

José Palomo *, José Rodríguez-Mirasol, and Tomás Cordero

Chemical Engineering Department, Andalucía Tech, Universidad de Málaga, 29010 Málaga, Spain
* Correspondence: palomo@uma.es; Tel.: +34-951-952-529

Received: 16 June 2019; Accepted: 5 July 2019; Published: 9 July 2019

Deconvolution of XPS spectra:

P2p:

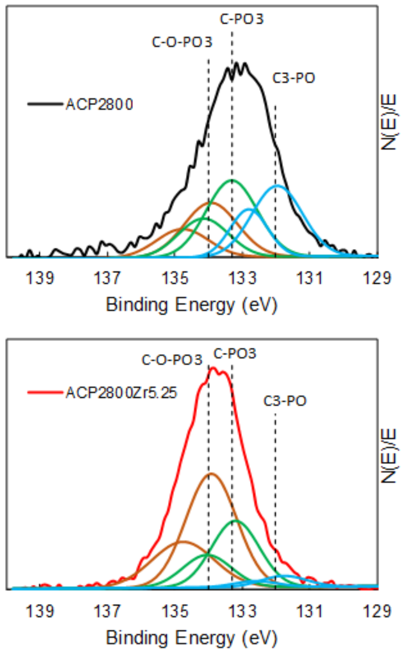

Figure S1. Deconvolution of the normalized P2p XPS spectra for ACP2800 and the 5.25 % Zr loaded phosphorus containing sample.

O1s:

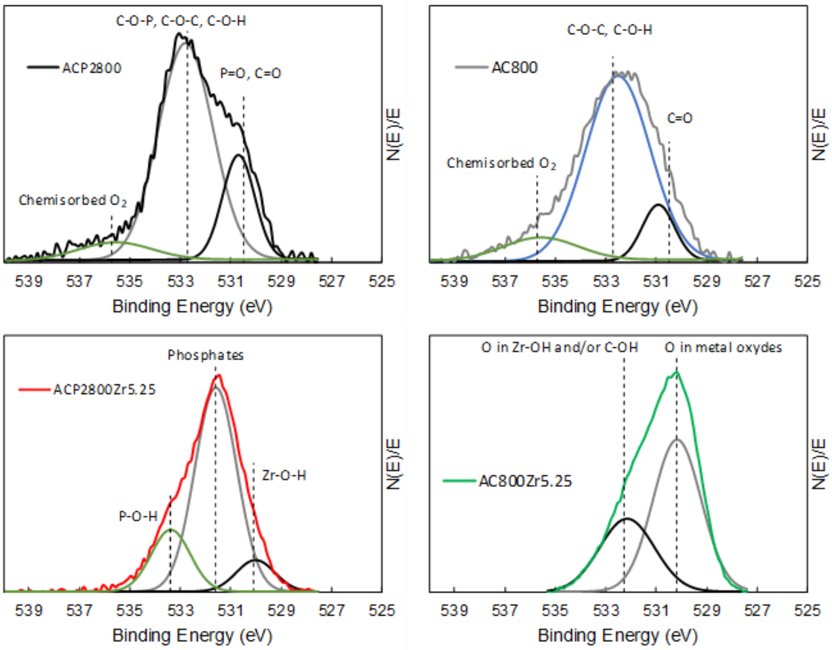

Figure S2. Deconvolution of the normalized O1s XPS spectra for ACP2800, AC800 and the 5.25 % Zr loaded samples (ACP2800Zr5.25 and AC800Zr5.25).

Zr3d:

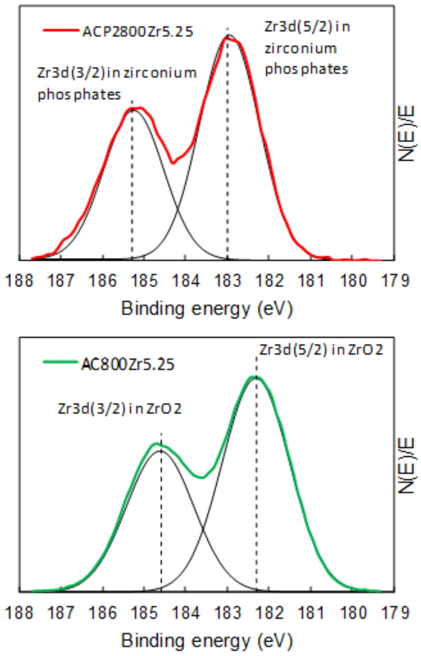

Figure S3. Deconvolution of the normalized Zr3d XPS spectra for the 5.25 % Zr loaded samples, ACP2800Zr5.25 and AC800Zr5.25.
